# The neuroprotective effect of cannabidiol is enhanced by resveratrol and alpha-lipoic acid in social isolation

**DOI:** 10.3389/fphar.2025.1676421

**Published:** 2025-10-29

**Authors:** Federica Ricciardi, Andrea Maria Morace, Rebecca Limongelli, Monica Iannotta, Serena Boccella, Antimo Fusco, Roozbe Bonsale, Michela Perrone, Rosmara Infantino, Emanuele Di Martino, Consalvo Mattia, Francesca Gargano, Maria Consiglia Trotta, Enza Palazzo, Sabatino Maione, Francesca Guida, Livio Luongo, Carmela Belardo

**Affiliations:** ^1^ Department of Experimental Medicine, University of Campania L. Vanvitelli, Naples, Italy; ^2^ Pharmacology and Therapeutics, School of Medicine, University of Galway, Galway, Ireland; ^3^ Centre for Pain Research, University of Galway, Galway, Ireland; ^4^ Galway Neuroscience Centre, University of Galway, Galway, Ireland; ^5^ Division of Anesthesiology, Intensive Care and Pain Medicine, ICOT Polo Pontino, Sapienza University of Rome, Rome, Italy; ^6^ Department of Medico Surgical Sciences and Biotechnologies, Sapienza University of Rome, Rome, Italy; ^7^ Department of Anesthesia and Resuscitation, Biomedical Campus University of Rome, Rome, Italy; ^8^ Department of Life Science, Health, and Health Professions, Link Campus University, Rome, Italy

**Keywords:** post traumatic stress disorder, cannabidiol, aggressiveness, antioxidant, behavior and social isolation

## Abstract

**Introduction:**

Post-traumatic stress disorder (PTSD) is a chronic psychiatric condition characterized by persistent cognitive and affective disturbances following exposure to severe trauma. In rodents, prolonged post-weaning social isolation is a well-established model of PTSD-like symptomatology. In this study, we investigated the behavioral effects of chronic cannabidiol (CBD) administration—either alone or in combination with two natural antioxidants, resveratrol (RES) and alpha-lipoic acid (ALA)in socially isolated mice.

**Methods:**

Male CD1 mice (n = 8) were isolated in individual cages from postnatal day 21 (PN21) and maintained in isolation for 30 days. They were then treated with CBD (2.5, 5, and 10 mg/kg), resveratrol (RES, 20 mg/kg), or alpha-lipoic acid (ALA, 10 mg/kg) for 15 days following social isolation.

**Results:**

While low-dose CBD (2.5 mg/kg) alone was ineffective, its combination with either RES or ALA restored the latency to the first attack and significantly reduced aggressive behavior, comparable to high-dose CBD (10 mg/kg). Similarly, combined treatments with RES or ALA markedly reduced immobility time in the tail suspension test, indicating antidepressant-like effects. In contrast, no significant anxiolytic effect was observed with the combinations in the hole-board test, suggesting a limited action on anxiety-like behavior.

**Discussion:**

These findings suggest that co-administration of CBD with RES or ALA exerts synergistic antidepressants and anti-aggressive effects in a PTSD-like model, potentially allowing for dose reduction of CBD. Further studies are warranted to explore the underlying molecular mechanisms.

## 1 Introduction

Post-traumatic stress disorder (PTSD) is a chronic psychiatric disorder characterized by persistent changes in mood, cognition, and arousal following exposure to severe traumatic events. According to the Diagnostic and Statistical Manual of Mental Disorders, 5th Edition (DSM-5), PTSD symptoms are classified into four main clusters: (i) intrusive symptoms (e.g., flashbacks, nightmares), (ii) negative alterations in mood and cognition, (iii) avoidance of trauma-related cues, and (iv) hyperarousal or hyperreactivity (e.g., irritability, concentration deficits) (Steenkamp, 2016; Pai et al., 2017). Although the exact pathophysiology of PTSD remains unclear, several neurobiological and psychosocial factors contribute to its development (Bailey et al., 2013). First-line treatments typically include psychotherapy combined with pharmacotherapy (e.g., selective serotonin reuptake inhibitors, serotonin-norepinephrine reuptake inhibitors, antiadrenergic agents, and second-generation antipsychotics) (Jeffreys et al., 2012). However, the efficacy of current medications remains limited, and side effects are common, underscoring the need for novel pharmacological strategies. Animal models are essential tools for elucidating disease mechanisms and testing therapeutic interventions. While rodents cannot fully recapitulate human PTSD, specific physiological and behavioral alterations mimic core features of the disorder. Prolonged post-weaning social isolation in mice is a widely used chronic psychosocial stress model relevant to several psychiatric conditions, including PTSD (Matsumoto et al., 2019; Pinna, 2019). Accumulating evidence supports the validity of this model, as it induces enduring behavioral and neurobiological changes that parallel PTSD symptomatology, such as heightened anxiety, cognitive deficits, social withdrawal, and hyperarousal (Berardi et al., 2016). These effects are underpinned by long-lasting alterations in stress-related brain circuits, including hypothalamic-pituitary-adrenal (HPA) axis dysregulation, impaired monoaminergic signaling, and disrupted prefrontal cortex-amygdala connectivity (Flandreau and Toth, 2017; Locci and Pinna, 2019; Vlachos et al., 2020). Notably, the model demonstrates strong face, construct, and predictive validity: it mirrors PTSD-like phenotypes, reflects etiological mechanisms (chronic social stress), and responds to conventional pharmacological treatments. Thus, it provides a reliable translational platform for investigating PTSD pathophysiology and novel therapeutic strategies.

Interestingly, we previously reported that in a weight-drop mild traumatic brain injury (TBI) mouse model which exhibits behavioral and neurobiological (Bonsale et al., 2023) features resembling PTSD cannabidiol (CBD) emerged as a novel pharmacological tool capable of restoring behavioral alterations and partially normalizing cortical biochemical changes (Belardo et al., 2019). CBD, a major non-psychoactive constituent of *Cannabis sativa*, has promising therapeutic potential for central nervous system (CNS) disorders (Schonhofen et al., 2018; De Gregorio et al., 2019). Through its multitarget mechanism of action, CBD demonstrates analgesic, anti-inflammatory, and antioxidant effects in preclinical models of neurodegenerative diseases and brain injury, such as hypoxia-ischemia (Carrier et al., 2006; Hayakawa et al., 2007; Castillo et al., 2010). In the present study, we investigated the effects of chronic CBD treatment on PTSD-like behavioral alterations linked to chronic oxidative stress, including social isolation-induced aggression, depressive-like behavior, and anxiety which is known as PTSD symptoms (Miller et al., 2018). Given that CBD’s anti-inflammatory and antioxidant properties vary by dose, route of administration, treatment duration, and experimental model may be insufficient to fully counteract PTSD-related oxidative stress (Hickey et al., 2024; Jîtcă et al., 2025), we tested its combination with two natural compounds: resveratrol (RES) and alpha-lipoic acid (ALA). These compounds have well-documented antioxidants and anti-inflammatory properties (Yan et al., 2011; He et al., 2012). This strategy aimed to achieve a synergistic neuroprotective effect while reducing the required CBD dosage and mitigating potential side effects, such as sedation and gastrointestinal disturbances (Chesney et al., 2020; Souza et al., 2022). Based on these premises, we hypothesized that combining subeffective doses of CBD with natural antioxidants (RES or ALA) would produce synergistic behavioral benefits in socially isolated mice. This approach could represent a promising pharmacological strategy to alleviate PTSD-related symptoms particularly aggression and depressive-like behavior while minimizing CBD dosage requirements.

## 2 Materials and methods

### 2.1 Animals

Male CD1 mice (Inotiv, Italy), weighing 16–18 g at study initiation, were used in this experiment. Animals were housed either individually (social isolation group) or in groups of 3-4 per cage (group-housed controls) under standardized laboratory conditions: 12-h light/dark cycle (lights on at 06:00), ambient temperature maintained at 20 °C–22 °C, and relative humidity of 55%–60%. All animals had *ad libitum* access to standard chow and tap water. Following a minimum 7-day acclimatization period, experimental procedures commenced. All experimental protocols were approved by the Animal Ethics Committee of the University of Campania “Luigi Vanvitelli”, Naples (Project Approval No. 24/2023-PR), in compliance with Italian (D.L. 116/92) and European (E.C. Directive 86/609/EEC) regulations for laboratory animal welfare. We implemented all necessary measures to minimize animal discomfort and optimize the experimental design to reduce the number of animals required. The health status of all animals was monitored throughout the entire study period to ensure both animal welfare and data integrity. This was performed through daily visual inspections by the research team and regular, formal health checks conducted by the institutional veterinary staff of the animal facility. Throughout the duration of the experiment, no signs of illness, distress, or other health-related complications were observed in any of the experimental groups.

### 2.2 Social isolation protocol and treatments

To induce a PTSD-like phenotype, a well-established model of prolonged post-weaning social isolation was used (Mumtaz et al., 2018). Mice were isolated in individual cages from postnatal day 21 (PN21) and maintained in isolation for 30 days. Group-housed mice served as controls.

**SCHEME 1:**
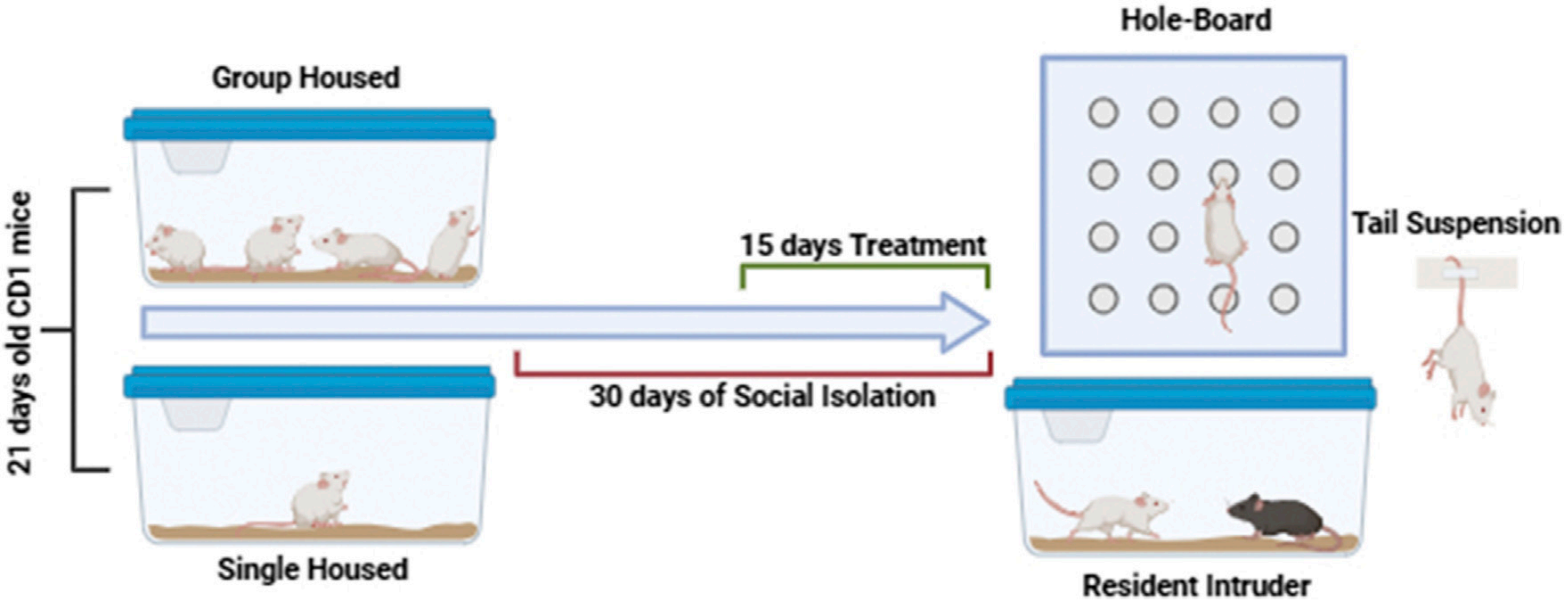
Timeline of social isolation, treatments and behavioral characterization.

Pharmacological treatments were administered via oral gavage (intragastric, i. g.), beginning 2 weeks after the onset of social isolation and continuing daily for 15 days, according to protocols described in previous studies (Guidotti et al., 2001; Pibiri et al., 2008; Locci and Pinna, 2019). All experiments were conducted by observers blinded to the treatment conditions. The experimental timeline is illustrated in Scheme 1. The cannabidiol dose (CBD) was selected based on previous studies demonstrating its efficacy in modulating neuroinflammatory and behavioral responses in murine models (Belardo et al., 2019) reported that CBD, administered intraperitoneally at 5 and 10 mg/kg, significantly reduced neuroinflammation and promoted neuroplasticity and functional recovery following spinal cord injury in mice. These findings support the relevance of using similar doses to explore the neuroprotective and behavioral effects of CBD in models of stress-related disorders.

Synthetic cannabidiol (CBD), resveratrol (RES), and alpha-lipoic acid (ALA) were generously provided by Crystal Hemp SA and were suspended in a vehicle for daily oral gavage. All treatments were administered at a standardized volume of 100 µL per mouse. The vehicle emulsion was prepared by 20% v/v sterile sesame oil with 80% v/v sterile saline (0.9% NaCl), stabilized with 2% v/v Tween 80; the mixture was homogenized for 2–3 min and then formed for 5–10 min to form a stable emulsion prior to each administration. The CBD doses were selected based on our previous study (Belardo et al., 2019), ALA based on studies demonstrating its efficacy in modulating oxidative stress (Dias-Ferreira et al., 2009; Araujo et al., 2017), and RES based on its documented neuroprotective and anti-inflammatory properties (Li et al., 2015; Wu et al., 2018).

### 2.3 Behavioral tests

Behavioral testing was conducted for all groups simultaneously, following the 30-day isolation period. To minimize animal stress, tests were spaced over consecutive days, allowing for recovery between sessions. All behavioral data in this study were collected and analyzed through direct observation and manual scoring. The scoring was performed by trained PhD students who are experienced in behavioral phenotyping. To ensure objectivity and minimize bias, the observers were blinded to the experimental groups of the animals during both the testing and the analysis phases. In the specific context of our study, which involved a social isolation stress paradigm, we deliberately omitted a formal habituation period to preserve the integrity of isolation and prevent the confounding effects of positive human interaction. This approach ensured that the measured behavioral outcomes were a direct result of the experimental condition.

#### 2.3.1 Aggressiveness: resident intruder test

CD1 mice were individually housed for 1 week in Plexiglas cages to establish home territory and to increase the aggression of the resident experimental miceTo begin, food containers were removed and an intruder mouse of the same sex but different strain (C57BL6/J, used as aggressor) was placed in a resident’s home cage, and resident-intruder interactions were analyzed for 10 min. The aggressive behavior of resident socially isolated mice was characterized by an initial pattern of exploratory activity around the intruder, which was followed by rearing and tail rattling, accompanied within seconds by wrestling and/or violent biting attacks. The number of attacks and latency to the first attack during the 10-min observation period were recorded (Belardo et al., 2019).

#### 2.3.2 Anxiety: hole board test

The hole board test is mainly used for assessing exploratory and social behaviors in rodents. The animal is placed in an arena with regularly arranged holes on the floor. Both frequency and duration of spontaneous elicited hole-poking behavior were measured during a short period of time. This test also provides a simple method for assessing anxious response of a rodent to an unfamiliar environment. The use of the hole-board in this perspective relies on the hypothesis that the behavior of animals exposed to a novel situation results from competition between an exploratory tendency and a withdrawal tendency. Thus, a high level of anxiety results in decreased head-dipping behavior, and inversely, a low level of anxiety manifests as increased head-dipping behavior. Other associated behaviors can be evaluated during the hole board test, such as grooming, rearing, and locomotion (Belardo et al., 2022).

#### 2.3.3 Depression: tail suspension test

This test has been used to evaluate depressive-like behavior. Mice were individually suspended by the tail from a horizontal bar (55 cm from the floor) using adhesive tape placed approximately 4 cm from the tip of the tail. The duration of immobility, recorded in seconds, was monitored during the last 4 min of the 6-min test by a time recorder. Immobility time was defined as the absence of escape-oriented behavior. Mice were considered immobile when they did not show any movement, hanging passively and completely motionless (Guida et al., 2018; Belardo et al., 2019).

### 2.4 Statistical analysis

The sample size for this study was determined based on established standards in the field of behavioral pharmacology experiments. A group size of n = 8 is commonly used and provides sufficient statistical power to detect meaningful effect sizes in paradigms such as the ones employed in this study. Data were analyzed using GraphPad Prism version 8.0 (GraphPad Software). Data were analyzed using one-way ANOVA, followed by Tukey’s multiple comparisons test to assess significant differences between groups. The effects of CBD were evaluated by constructing dose-effect curves, with logED50 values and 95% confidence limits calculated by GraphPad Prism 8.0. The percentage of the maximum possible effect for each experiment was quantified and calculated as [(experimental value - baseline value)/(max cutoff - baseline value)] × 100.

## 3 Results

### 3.1 Effect of CBD alone or in combination with resveratrol or alpha lipoic acid on the aggressive behavior induced by social isolation

**FIGURE 1:**
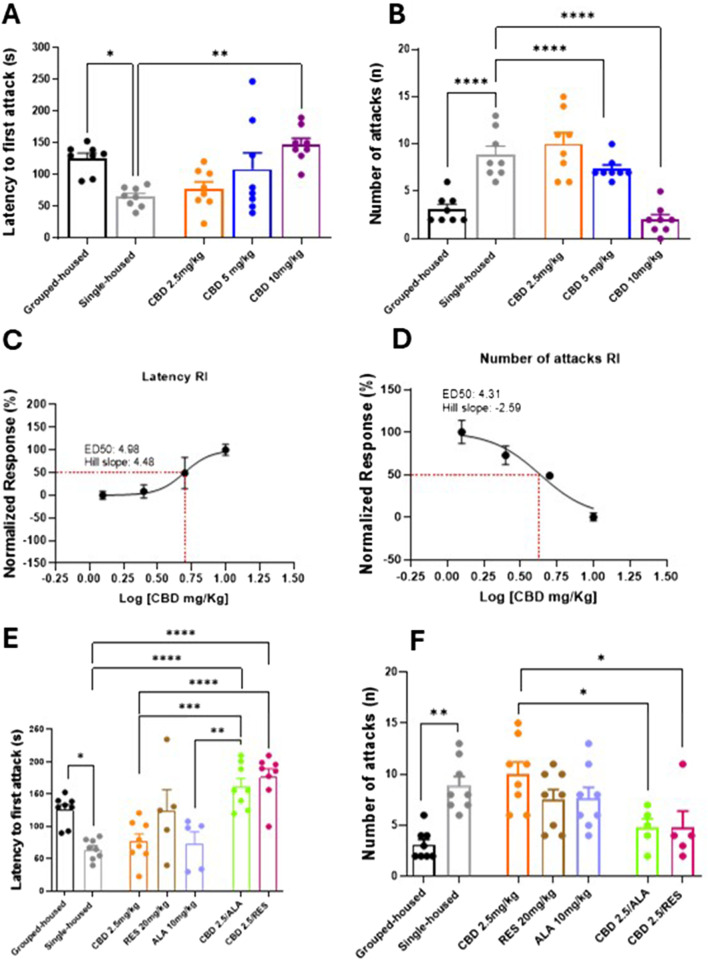
Effect of CBD **(A,B)** at different doses or Resveratrol, Alpha lipoic acid alone or in combination with lowest dose of CBD (2.5 mg/Kg) **(E,F)** in the resident intruder test. Each time point represents the Mean ± SEM of 8 mice per group. **(C,D)** Dose-response curve showing ED50 (4.96 mg/Kg and 4.31 mg/Kg, respectively) and Hill slope (4.48 and −2.59, respectively) of CBD on the latency to first attack and on the number of attacks in RI. The results are expressed as logarithmic doses of CBD relative to the percentage of normalized response. Data were analyzed by using one-way ANOVA, followed by Tukey’s multiple comparisons test. P < 0.05 was considered statistically significant.

Aggressive behavior was assessed using the resident-intruder test. Socially isolated mice exhibited a significantly decreased latency to first attack and an increased frequency of attacks compared to group-housed controls (Figures 1A,B). (Latency: 65.0 ± 5.58 vs. 126.0 ± 7.97; Number of attacks: 8.87 ± 0.89 vs. 3.12 ± 0.51). Chronic oral administration of CBD (2.5, 5, and 10 mg/kg) after 14 days significantly and dose-dependently increased the latency of the first attack (Latency: CBD 10 mg/kg 147.5 ± 9.95; CBD 5 mg/kg 108.4 ± 26.11; CBD 2.5 mg/kg 77.50 ± 11.20; F_(4, 35)_ = 18,58; P = 0,0011) and reduced the frequency of attacks in single-housed mice (Number of attacks: CBD 10 mg/kg 2.00 ± 0.53; CBD 5 mg/kg 7.37 ± 0.41; CBD 2.5 mg/kg 10.00 ± 1.19; F_(4, 35)_ = 21,15; P < 0,0001) (Figures 1A,B). CBD reduced the aggression in a dose-response manner (Figures 1C,D). Notably, the combination of a sub-effective dose of CBD (2.5 mg/kg) with either RES (20 mg/kg) or ALA (10 mg/kg) significantly enhanced the latency of the first attack (Latency: CBD 2.5 mg/kg 77.50 ± 11.20; ALA 10 mg/kg 74.20 ± 17.32; RES 20 mg/kg 125.2 ± 31.83; CBD 2.5 mg/kg-ALA 10 mg/kg 162.1 ± 12.13; CBD 2.5 mg/kg-RES 20 mg/kg 177.0 ± 12.33; F_(6, 43)_ = 11.57; P < 0,0001) and reduced the number of attacks (Number of attacks: CBD 2.5 mg/kg 10.00 ± 1.19; ALA 10 mg/kg 7.62 ± 10.85; RES 20 mg/kg 7.5 ± 1.00; CBD 2.5 mg/kg-ALA 10 mg/kg 4.8 ± 0.86; CBD 2.5 mg/kg-RES 20 mg/kg 4.8 ± 1.59; F_(6, 43)_ = 5.98; P = 0001) compared to the compound alone (Figures 1E,F). Taken together, these results demonstrate that CBD exerts a dose-dependent anti-aggressive effect, which is significantly potentiated by combination with either resveratrol or alpha-lipoic acid.

### 3.2 Effect of CBD alone or in combination with resveratrol or alpha lipoic acid on depressive-like behavior induced social isolation

**FIGURE 2:**
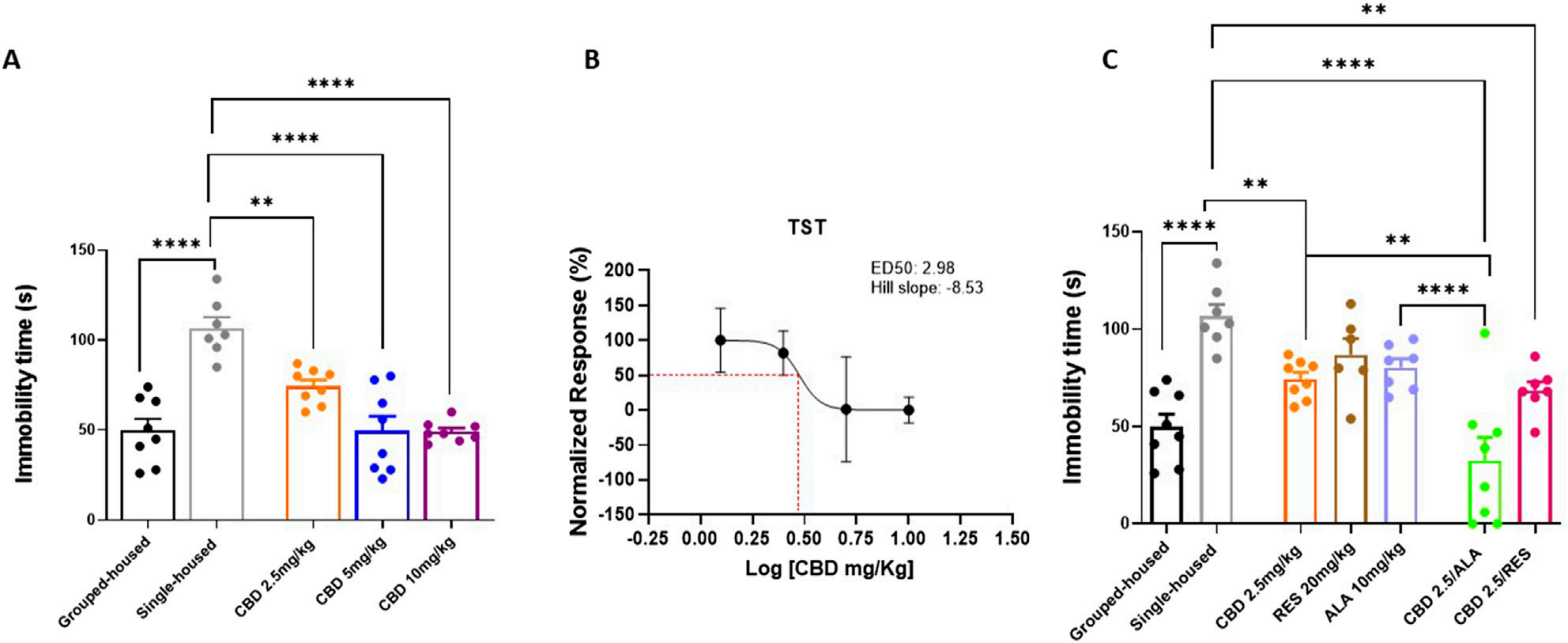
Effect of CBD** (A)** at different doses or Resveratrol, Alpha lipoic acid alone or in combination with lowest dose of CBD (2.5 mg/Kg) **(C)** in the tail suspension test. Each time point represents the Mean ± SEM of 8 mice per group. **(B)** Dose‐response curve showing ED50 (2.98 mg/Kg) and Hill slope (−8.53). The results are expressed as logarithmic doses of CBD relative to the percentage of normalized response. Data were analyzed by using one-way ANOVA, followed by Tukey’s multiple comparisons test. P < 0.05 was considered statistically significant.

In the Tail Suspension Test, used to assess depressive-like behavior, behavioral responses were quantified as immobility time (seconds). Single-housed mice showed a significant increase in immobility duration, measured by the absence of escape-oriented behavior, compared to group-housed mice (Figures 2A) (106.7 ± 6.042; 49.88 ± 6.44). Repeated oral administrations of CBD significantly reduced the immobility time (CBD 10 mg/kg 49.13 ± 2.03; CBD 5 mg/kg 49.50 ± 8.20: CBD 2.5 mg/kg 74.38 ± 3.48; F _(4, 34)_ = 18,58; P < 0,0001) (Figures 2A,B). Interestingly, the combination of lowest dose of CBD (2.5 mg/kg) with ALA (10 mg/kg) or RES (20 mg/kg) induced a significant decrease of the immobility time in isolated mice in comparison to the group housed animals (CBD 2.5 mg/kg 74.38 ± 3.49; ALA 10 mg/kg 80.43 ± 4.37; RES 20 mg/kg 86.83 ± 8.38; CBD 2.5 mg/kg-ALA 10 mg/kg 32.50 ± 11.86; CBD 2.5 mg/kg-RES 20 mg/kg 68.57 ± 4.42; F_(6, 44)_ = 12,05; P < 0,0001) (Figures 2C). Collectively, these data confirm the efficacy of CBD against depressive-like behavior and reveal a synergistic therapeutic benefit when it is combined with alpha-lipoic acid.

### 3.3 Effect of CBD alone or in combination with resveratrol or alpha lipoic acid on the anxiety-like behavior induced by social isolation

**FIGURE 3:**
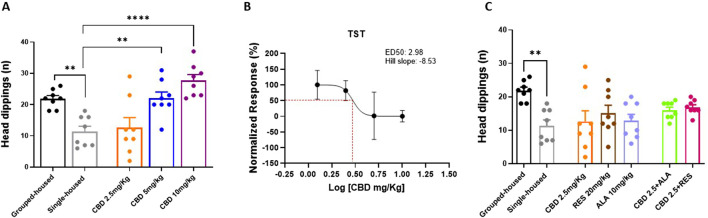
Effect of CBD **(A,C)** at different doses or Resveratrol, Alpha lipoic acid alone or in combination with lowest dose of CBD (2.5 mg/Kg) in the hole board test. **(B)** Dose‐response curve showing ED50 (3.29 mg/Kg) and Hill slope (2.98) of CBD. The results are expressed as logarithmic doses of CBD relative to the percentage of normalized response. Each time point represents the Mean ± SEM of 8 mice per group. Data were analyzed by using one‐way ANOVA, followed by Tukey’s multiple comparisons test. P < 0.05 was considered statistically significant.

In the Hole Board Test, used to assay general anxiety, behavioral skills were quantified as number of head dippings. Single-housed mice showed a significant reduction in the number of head dippings as compared to grouped-house mice (11.38 ± 1.731; 21.88 ± 1.025). Repeated oral administrations of CBD increased the number of head dippings in a dose-response manner (5 and 10 mg/kg) in single-housed mice (CBD 10 mg/kg 27.75 ± 1.84; CBD 5 mg/kg 22.00 ± 2.02; CBD 2.5 mg/kg 12.63 ± 3.20; F_(4, 35)_ = 11,03; P < 0,0001) (Figures 3A,B). No effects were observed in single-housed mice treated with CBD (2.5 mg/Kg), RES, ALA alone or in combination with CBD (2.5 mg/kg) (CBD 2.5 mg/kg 12.63 ± 3.20; ALA 10 mg/kg 12.88 ± 1.88; RES 20 mg/kg 15.13 ± 2.34; CBD 2.5 mg/kg-ALA 10 mg/kg 16.00 ± 0.90; CBD 2.5 mg/kg-RES 20 mg/kg 16.88 ± 0.76; F _(6, 49)_ = 3,516; P = 0,0057) (Figures 3C). These results demonstrate that CBD produces a dose-dependent anxiolytic effect in the Hole Board Test, but this effect is not observed with a low dose of CBD alone or when it is combined with either resveratrol or alpha-lipoic acid.

## 4 Discussion

The key finding of this study demonstrates the efficacy of CBD combined with either resveratrol or ALA in counteracting affective disturbances induced by chronic social isolation in mice. Isolated mice exhibited neuropsychiatric alterations characterized by an anxious-depressive state accompanied by aggressive behavior. This co-occurrence of depressive and aggressive behaviors mirrors clinical observations in PTSD patients, where major depressive episodes often alternate with aggressive outbursts (Lyketsos et al., 1999; Maj et al., 2003; Taft et al., 2005). Prolonged social distancing, as experienced during the COVID-19 pandemic, weakens social bonds and leads to significant psychophysical consequences (Clemente-Suárez et al., 2020). Preclinical studies investigating isolation-induced psychopathology suggest that these affective changes may involve endocannabinoid system dysregulation in limbic and cortical regions highlighting the potential therapeutic value of modulating endocannabinoid tone (Brugnatelli et al., 2021).

Consistent with previous reports (Belardo et al., 2019; Hartmann et al., 2019), our results show that repeated CBD treatment alleviates anxiety, depression, and aggression inzz isolated mice. Combining subeffective CBD doses with either resveratrol or ALA produced significant behavioral improvements, suggesting synergistic interactions. We specifically employed oral gavage administration to enhance translational relevance, as this route closely parallels the clinical administration of nutraceuticals and cannabinoid-based formulations in humans.

This study was conducted exclusively on male mice as a pilot experiment to characterize preliminary neurobehavioral outcomes before expanding the investigation to both sexes. Although this approach reduced variability linked to hormonal fluctuations, it constitutes a limitation, and the possible sex-related effects remain to be determined (Hartmann et al., 2019). demonstrated that a single injection of CBD (10 mg/kg) can modulate aggressive behavior in isolated mice, an effect which was hypothesized to be mediated by the 5-HT1A and CB1 receptor activation. Our findings, showing efficacy of a low, repeated CBD dose, align with evidence underscoring the therapeutic potential of low-concentration regimens. In a chronic unpredictable mild stress model, a very low dose of CBD (1 mg/kg) reversed stress-induced behavioral despair and restored excitatory synapses in the medial prefrontal cortex, normalizing the number and size of VGlut1 and F-actin positive synaptic clusters and enhancing their colocalization. Proteomic data supported these findings, indicating CBD’s role in restoring glutamatergic and serotonergic pathway integrity (Sales et al., 2019; Reuveni et al., 2022; Borràs‐Pernas et al., 2025). This provides a neurobiological correlation for our observations, suggesting that the subeffective CBD dose (2.5 mg/kg) used in our study may reverse synaptic deficits and network imbalances induced by chronic social isolation.

Our results confirm CBD’s efficacy in reducing aggression and show that combining a subeffective CBD dose (2.5 mg/kg) with either resveratrol or ALA completely normalized both attack latency and frequency in isolated mice, achieving effects comparable to high-dose CBD. Similarly, we observed reduced immobility time during the tail suspension test in mice treated with ALA + CBD or RES + CBD compared to those receiving CBD alone. The selection of resveratrol and alpha-lipoic acid (ALA) was based on their complementary mechanisms: resveratrol modulates inflammatory signaling and SIRT1-mediated neuroprotection (Shin et al., 2012), while ALA functions as a mitochondrial antioxidant and glutathione recycling modulator (Superti and Russo, 2024).

The efficacy of resveratrol in our study is supported by findings from Tabassum et al. (Tabassum et al., 2023), who demonstrated in a chronic unpredictable mild stress model that resveratrol reversed anxiety and depressive-like behaviors through the SIRT1/PGC1α/SIRT3 signaling pathway in the medial prefrontal cortex. Resveratrol normalized mitochondrial dynamics, restored ultrastructural integrity, and reversed the loss of PV + GABAergic interneurons, restoring inhibitory tone. In our context, resveratrol likely synergized with CBD by targeting mitochondrial and GABAergic dysfunction in the PFC.

Similarly, ALA’s efficacy in mitigating neuropsychiatric outcomes is supported by evidence showing its antioxidant and anti-inflammatory effects in the prefrontal cortex and hippocampus, where it restored glutathione levels, reduced lipid peroxidation, lowered IL-1β, and elevated brain-derived neurotrophic factor (BDNF) concentrations (Gomes et al., 2025). These mechanisms may underlie the observed behavioral synergism with CBD, as ALA’s ability to normalize redox balance and boost BDNF could facilitate CBD’s prosocial and antidepressant effects. The doses used (RES: 20 mg/kg; ALA: 10 mg/kg) were derived using allometric scaling and intentionally maintained below clinical equivalence to assess synergistic potential with CBD. When combined with CBD’s effects on serotonergic and endocannabinoid systems, these antioxidants may promote neuroplasticity, reduce neuroinflammation, and restore oxidative balance.

The observed behavioral improvements with CBD and antioxidant co-administration are supported by CBD’s known neuroprotective and neuroplastic effects. Chronic CBD administration counteracts hippocampal neurogenesis reduction, dendritic spine loss, and microglia activation while protecting PV + interneurons, key pathological features of anxiety and depression (Campos et al., 2017). CBD facilitates endocannabinoid signaling, acts as a 5-HT1A receptor modulator, and activates neuroprotective pathways such as autophagy and PPARγ. Thus, RES and ALA likely potentiate CBD’s neuroprotective profile by further reducing oxidative and inflammatory stress, enhancing neuroplastic recovery.

Although mechanistic pathways were not investigated, we cannot exclude that treatments may elevate BDNF or enhance serotonin neurotransmission in hippocampal and cortical regions, as previously reported (Zanelati et al., 2010; Gu et al., 2019). The impact of CBD on BDNF appears context-dependent. Some studies report no change in total hippocampal BDNF after acute or sub-chronic treatment (Zanelati et al., 2010; Campos et al., 2017), but this may result from methodological factors such as measuring total rather than localized BDNF pools. Notably, CBD’s rapid antidepressant-like effects have been linked to increased extracellular serotonin and glutamate in the ventromedial prefrontal cortex, an effect blocked by 5-HT1A antagonism (Campos et al., 2017). This suggests that CBD’s benefits may stem from rapid modulation of monoaminergic and glutamatergic transmission in corticolimbic areas.

Divergent effects were found for anxiety-related behaviors. Chronic CBD treatment produces anxiolytic effects in humans (Elsaid et al., 2019) and in animal models including chronic unpredictable stress (Fogaça et al., 2018) and early-life stress (Martín-Sánchez et al., 2022), effects largely attributed to CB1 receptor indirect activation (Austrich-Olivares et al., 2022). Consistent with this, our highest CBD dose reduced head-dipping behavior in the hole board test. However, low-dose CBD and its combinations with RES or ALA showed no significant anxiolytic effects in our model. This may reflect the high sensitivity of the hole board test to novel elevated environments, where strong stress responses can mask subtle anxiolytic effects. The lack of synergy suggests that CBD-antioxidant combinations may not affect anxiety-related neural circuits at these doses or may require longer treatment duration.

In conclusion, this study demonstrates that CBD combined with resveratrol or ALA effectively reduces aggression and depression-like behaviors under chronic social isolation. These combinations may represent promising pharmacological strategies for managing PTSD-related affective disturbances while allowing CBD dose reduction. Future work should investigate underlying molecular mechanisms, sex differences, and neurobiological correlates of CBD-antioxidant synergism.

## Data Availability

The original contributions presented in the study are included in the article/supplementary material, further inquiries can be directed to the corresponding author.
